# Medical Obedience

**Published:** 1846-04

**Authors:** 


### MEDICAL OBEDIENCE.

So the introductory lecture of Professor Darrach, of the Pennsylvania Medical College, is entitled. Obedience to the command, "*Go heal the sick,*" is the subject of his lecture—an earnest, poetical, pious discourse, written in a hurried conversational style, but full of statistics, anecdotes, and just reflections. We give a specimen: "On a visit of respect to Scarpa, in 1821, he told me that he had been constantly in the labor of his profession of 62 years, and that he still loved it. He was then at the age of 80 years, and had just published a folio volume of observations and recent experiments on the proper method of tying arteries. And, said he, in the course of conversation, at 18 years of age I, on a medical tour, was the means of introducing the use of the catheter from Paris into London, and the practice of dispensing with the charpee from London into Paris."

